# Bio::NEXUS: a Perl API for the NEXUS format for comparative biological data

**DOI:** 10.1186/1471-2105-8-191

**Published:** 2007-06-08

**Authors:** Thomas Hladish, Vivek Gopalan, Chengzhi Liang, Weigang Qiu, Peter Yang, Arlin Stoltzfus

**Affiliations:** 1 Center for Advanced Research in Biotechnology, 9600 Gudelsky Drive, Rockville, MD 20850, USA; 2 Section of Integrative Biology, University of Texas at Austin, 1 University Station C0930, Austin, Texas 78712, USA; 3 Cold Spring Harbor Laboratory, 1 Bungtown Rd, Cold Spring Harbor, NY 11724, USA; 4 Department of Biological Sciences, Hunter College, CUNY, 695 Park Ave., New York, NY 10021, USA; 5 Biochemical Science Division, National Institute of Standards and Technology, Gaithersburg, MD 20899-8310, USA

## Abstract

**Background:**

Evolutionary analysis provides a formal framework for comparative analysis of genomic and other data. In evolutionary analysis, observed data are treated as the terminal states of characters that have evolved (via transitions between states) along the branches of a tree. The NEXUS standard of Maddison, et al. (1997; *Syst. Biol*. 46: 590–621) provides a portable, expressive and flexible text format for representing character-state data and trees. However, due to its complexity, NEXUS is not well supported by software and is not easily accessible to bioinformatics users and developers.

**Results:**

Bio::NEXUS is an application programming interface (API) implemented in Perl, available from CPAN and SourceForge. The 22 Bio::NEXUS modules define 351 methods in 4229 lines of code, with 2706 lines of POD (Plain Old Documentation). Bio::NEXUS provides an object-oriented interface to reading, writing and manipulating the contents of NEXUS files. It closely follows the extensive explanation of the NEXUS format provided by Maddison et al., supplemented with a few extensions such as support for the NHX (New Hampshire Extended) tree format.

**Conclusion:**

In spite of some limitations owing to the complexity of NEXUS files and the lack of a formal grammar, NEXUS will continue to be useful for years to come. Bio::NEXUS provides a user-friendly API for NEXUS supplemented with an extensive set of methods for manipulations such as re-rooting trees and selecting subsets of data. Bio::NEXUS can be used as glue code for connecting existing software that uses NEXUS, or as a framework for new applications.

## Background

### Evolutionary comparative analysis

In comparative biology, inferences are made from patterns of similarities and differences. Contemporary genome analysis relies heavily on such comparisons, e.g., as in the way that human genome annotation has relied on comparisons with the mouse and chimp genomes. Evolutionary biologists have developed a specialized methodology for comparative analysis that draws on modern methods of statistical inference and that is (in principle) widely applicable to all sorts of biological data – from molecular sequences and protein activities to morphologies and behaviours [1].

The evolutionary approach provides a framework to convert problems of how to analyze similarities and differences into well posed questions about rates of various possible evolutionary transitions along the branches of a tree (phylogeny). As genome analysis and other systematic types of comparative analysis mature, and researchers seek to extract the maximum amount of useful information from available data, these methods of "evolutionary comparative analysis" have become increasingly important [2,3].

### The character-state data model

The methodological generality of evolutionary analysis relies on what we refer to here as "the character-state data model", a kind of Entity-Attribute-Value model. In the character-state data model, observations or measurements on a set of entities called "OTUs" (Operational Taxonomic Units) are represented as the observed values, called "states" (or "character states"), of a set of underlying attributes, called "characters". For a protein sequence alignment, for instance, each OTU (entity) is a protein, the homologous characters (attributes) are the alignment columns, and the state (value) of each OTU for a particular character is an amino acid or a gap (the latter often represented with the symbol "-"). Characters for which the states are finite are discrete characters; continuous characters are also possible (e.g,. beak length, protein activity). In biology, data sets often are incomplete, thus the state of a character may be denoted as "missing" (often represented with the symbol "?").

The assignment of characters is based on homology indicative of common ancestry. The presence of different states for the same (ancestral) character thus implies historical changes (transitions of state) from a common ancestral state. The paths of change are not all independent, but follow the branchings of a phylogenetic tree. That is, in evolutionary analysis, hierarchical clustering of data is not merely a heuristic tool, but reflects a generative model, an explicit or implicit model of character-state transitions that take place along the branches of a tree. The simplest model is that any change from one state to another observed state has the same probability or cost. More complex models may introduce restrictions on allowed transitions, or introduce non-uniform costs for possible transitions, or may relate the rates of transitions to a mechanistically inspired model (e.g., separate rate parameters for synonymous or non-synonymous changes).

### The NEXUS format

**Figure 1:**
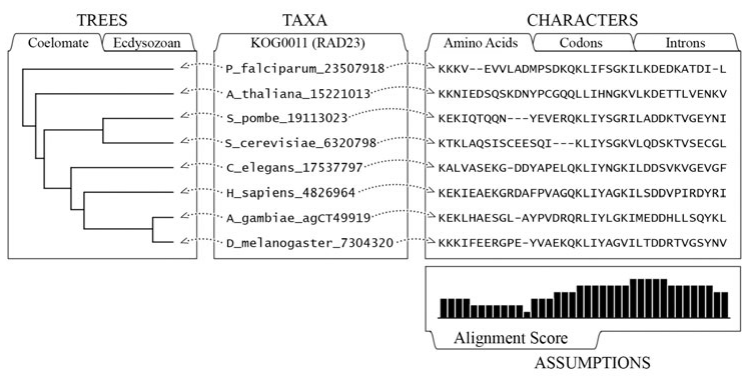
**NEXUS and the character-state data model**. Some relevant terms and concepts are illustrated with a graphical view of a small family of RAD23-related protein-coding genes (KOG0011 data provided in Additional file 1). In molecular sequence analyses, OTUs often are labelled with a token fusing a species name with a database ID. In a NEXUS file, such OTU labels are declared in a TAXA block. The TREES block may contain one or more trees relating the OTUs, each tree optionally having label (e.g., "Coelomate") and a numeric weight (e.g., a probability). Trees may contain branch lengths and support values. In the matrix of amino acid character data shown here, the 4^th ^character (i.e., the 4^th ^alignment column), has the states "V", "I", "I", "L", "V", "I", "L", and "I". Such character-state data are stored in a NEXUS CHARACTERS block, which defines the type of data and the meaning of a gap symbol such as "-". The ASSUMPTIONS block of a NEXUS file provides the means to store a weight or other numeric value for each character, such as the column-wise alignment scores shown here. Many other types of information not shown here can be stored in NEXUS files.

NEXUS [4] is a data exchange file format for character-state data and trees, used in software such as Mesquite [5], PAUP* [6], and MrBayes [7]. The terminology used in NEXUS files, and in the NEXUS standard, draws on the implicit character-state data model, e.g., columns of comparative data are referred to as "characters" (e.g., they are stored in a "CHARACTERS" block, within which labels are assigned via the "charlabels" command), for which each OTU has a "state". Note, however, that the OTUs in a NEXUS file are referred to as "taxa" (thus, a "TAXA" block has a "taxlabels" command) rather than as "OTUs". Figure 1 depicts the relationship between an example NEXUS dataset (Additional file 1) and the character-state-data model.

While the name "NEXUS" is capitalized by convention, NEXUS keywords are (with few exceptions) case-insensitive. However, for purposes of clarity we capitalize the keywords used to designate NEXUS blocks, such as TAXA, CHARACTERS, and TREES.

Although NEXUS files initially were used mainly for morphological data, the developers of NEXUS allowed for diverse types of data, including provisions for such things as frequency distributions of states (for each OTU), as well as commands specific for molecular data, so as to specify codon locations and to define alternative genetic codes. As a result, NEXUS continues to provide a rich way to represent diverse types of data that might be used in a comparative analysis of genes, proteins, or genomes. NEXUS files often include several types of information, the most common being:

• a discrete character matrix (e.g., a molecular sequence alignment)

• a continuous character matrix

• one or more rooted or unrooted phylogenies, each with (optional) name and weight

• a distance matrix

• a description of constraints on, or costs of, transitions between states of characters

• other assumptions such as weights for characters

Within a NEXUS file, data are organized into blocks, such that (in general) one block corresponds to one type of data. The complete list of blocks is given (in order of importance) in Table 1. The most commonly used blocks are TAXA (to define the set of OTUs), CHARACTERS (defining a set of character data) and TREES. The use of a DATA block – essentially a CHARACTERS block for which the OTUs have not been pre-defined via a TAXA block – is deprecated.

**Table 1:** NEXUS blocks and common commands

**Block name**	**Commonly used commands**	**Typical content of block**
**TAXA**	dimensions, taxlabels	Labels for OTUs (may be quoted strings)
**CHARACTERS**	dimensions, format, matrix	A matrix of character-state data (e.g., aligned residues) of defined type, in a defined format
**TREES**	tree, translate	Rooted or unrooted trees in Newick format, with optional names and weights for each tree
**ASSUMPTIONS**	options, usertype, wtset	Constraints on allowed changes; transition cost model; weights for characters
**CODONS**	codonposset, geneticcode	Description of a reading frame and the genetic code used to translate it
**SETS**	charset, taxset, treeset	Named sets of OTUs (taxsets), trees, characters, states, or state transitions (changesets)
**NOTES**	text, picture	Annotations attaching text or pictures to sets of objects including OTUs, characters or trees
**DISTANCES**	dimensions, format, matrix	Distance matrices
**UNALIGNED**	dimensions, format, matrix	Unaligned data
**DATA**^1^	(see CHARACTERS)	OTU labels and character data (deprecated)

^1 ^The DATA block format, though commonly used, is deprecated so as to encourage use of a separately declared list of OTUs in a TAXA block.

Within a block, information is organized into commands and their arguments, where arguments may either be data or subcommands that refine the scope of the command. For example, a CHARACTERS block could contain the three command-argument pairs shown below, each ending with a semi-colon:

BEGIN CHARACTERS;

   DIMENSIONS nchar=12;

   FORMAT datatype=protein gap=-;

   MATRIX

      Taxon1 ISPTCAP--RSV

      Taxon2 EAPKCAPGVGLV

      Taxon3 EAPKCAPGV-LV

      Taxon4 QKPRCPPGVSLV ;

END;

A NEXUS file seems well suited for use as both input and output for the analysis of a single coherent data set with an evolutionary history. For example, it would be appropriate to use a single NEXUS file to store all of the data associated with a set of tubulin genes that are to be analyzed together in a family-wide analysis, including sequences, species source, expression data, activity data, and so on. However, if one is studying both the tubulin gene family and the actin gene family, two NEXUS files are appropriate.

The original NEXUS standard provides for extensibility via "private" application-specific blocks. This idea, as successfully used in programs such as PAUP* [6], is that all of the commands necessary for batch processing of a file can be added to the file itself in a private application-specific block (in the case of PAUP*, there is a "PAUP" block with commands that only PAUP can understand).

### Benefits of an open-source Perl interface to NEXUS

The NEXUS file format is used by several programs or packages that molecular evolutionists find useful, including Mesquite [5], MrBayes [7], and HyPhy [8]. As evolutionary analysis becomes increasingly common in bioinformatics workflows, the need will grow for formats such as NEXUS that store data and trees together.

More importantly, the scope of evolutionary analysis – not merely the number of users – continues to expand. That is, while the mainstays of evolutionary analysis in the past were molecular sequence characters and discrete morphological characters, in recent years evolutionary methods have been applied to the analysis of data on genomic gene content [9], gene expression [10], and even "function", both at the level of a whole gene (or protein) [2] and at the level of individual sites within a protein [3]. Thus, it is significant that the NEXUS file implements (implicitly) a very flexible entity-attribute-value model (the character-state data model), which means that it can be applied to diverse types of data.

Existing software that uses NEXUS is mostly embedded in applications code such as PAUP* [6]. Some exceptions are the NEXUS methods provided by open-source toolkits such as BioPerl [11], and by NCL [12], for "NEXUS Class Library", a special purpose library written in C++. Most such implementations provide limited coverage of the NEXUS standard, and focus only on input and output of files, without providing a toolbox of methods. More complete implementations of NEXUS that provide a richer set of functions are needed, as in the Java source code included with Mesquite [5]. A Perl implementation would be valuable due to the popularity of Perl with biological programmers, who value its ease of use.

## Implementation

### Object-oriented Perl practices

Bio::NEXUS is written as a set of object-oriented (OO) Perl modules. An OO interface is ideal for two major reasons. First, NEXUS files are syntactically and organizationally complex, and OO Perl provides an easy-to-use abstraction. Users, for example, can rename taxa throughout a NEXUS file by using the *rename_otus *method and providing a map of old names to new names, without worrying about the details of how that object is structured or where the names are stored. By allowing Bio::NEXUS to handle the file formatting, users can focus on manipulating and interpreting data. Second, An OO implementation is also useful when data can be organized hierarchically. For example, a Bio::NEXUS::Tree (a phylogeny) is part of a Bio::NEXUS::TreesBlock, which is a Bio::NEXUS::Block, which is part of a Bio::NEXUS object. In this way, all block types can inherit generic functions (methods) from the Bio::NEXUS::Block class. The OO structure also means that a Bio::NEXUS object "knows" what it comprises, so that when a method is called (such as to remove some OTUs), the API automatically calls all the necessary methods at lower hierarchical levels (such as removing the OTUs from the character matrices, the trees, and so on).

The Bio::NEXUS API contains 22 modules in 22 files, organized in a way that reflects the structure of Bio::NEXUS objects: within the "Bio" directory (at the top level of the library) are the NEXUS base package and a directory called "NEXUS", which in turn contains the remaining 21 packages that define parts of the NEXUS class. In most cases, these 21 packages represent different block types, although there are some exceptions, e.g. there are separate tree and node classes that allow tree methods to be separated from the complexities of NEXUS files.

The 22 Bio::NEXUS modules define 351 methods, of which 250 are public methods, meaning they provide the API. The modules contain 4229 lines of code, 2706 lines of POD (Plain Old Documentation), and 391 lines of embedded comments for a total of 7326 non-whitespace lines.

### NEXUS parsing and the NEXUS standard

NEXUS files may be thought of as a series of commands, where each command ends with a semicolon:

<command_word> [<subcommand>] [<arguments>];

The one exception is that NEXUS files must begin with the string '#NEXUS', without quotation. NEXUS commands may be as simple as 'END;', which is used to indicate the end of a block, or as complex as the MATRIX command, which can take an entire multiple sequence alignment as its argument list. The Bio::NEXUS parser reads in an entire NEXUS file and breaks it into tokens, thereby making whitespace unimportant except as a delimiter. Spaces, tabs, and newlines are treated equivalently as delimiters, and multiple consecutive whitespace characters are treated as one. It is therefore possible for an entire NEXUS file, including a multiple sequence alignment and tree, to be on one line. The rare exception is that users who wish to store an interleaved multiple sequence alignment must use newlines to denote the end of each alignment row.

Interpreting the NEXUS file format requires a sophisticated, context-sensitive parser. In the NEXUS standard, a token is defined as a word (an unbroken sequence of non-punctuation, non-whitespace characters) or a single punctuation character (()[]{} /\, ; : = * ' ” ' + - < >). Single or double quotation marks, however, cause the enclosed to be treated as a single 'word', such as 'Human (alpha tubulin) [20–94]'. The underscore is taken to be synonymous with a single space; therefore, 'Human alpha tubulin' and Human_alpha_tubulin are indistinguishable to the Bio::NEXUS parser, although the latter format is always used for output.

When not quoted, square brackets denote comments, therefore, the string

   Human (alpha tubulin) [20–94]

if unquoted, would be interpreted as three words and one comment. Comments may be nested, as in [this [example]], in which case all square brackets must be paired, although nested comments have no special meaning per se. Bio::NEXUS attempts to keep track of the location of comments and reassociate them with the correct block when writing output. However, as Bio::NEXUS can be used to add, remove, and alter blocks or commands, it may be impossible to place a comment adjacent to its original command or block. For this reason (and others), comments should not be used as an *ad hoc *method of storing data.

The NEXUS format often is extended by application-specific or "private" blocks as allowed in the original format description [4]. If the Bio::NEXUS parser encounters an unfamiliar block type (e.g., a private block generated by MrBayes or PAUP*), an UnknownBlock object is created that stores (verbatim) the content of the unfamiliar block; this content is then included appropriately in the output stream whenever the Bio::NEXUS object is written out.

If the parser encounters an unfamiliar command (within a familiar type of block), however, an error results. This behavior is desirable for two reasons. First, unfamiliar commands often represent syntax errors or misspellings. Second, commands may be used to change how data are interpreted, thus ignoring an unfamiliar command without throwing an error could mislead users to assume that the data have been interpreted correctly.

### Structure of Bio::NEXUS objects

The structure of Bio::NEXUS objects reflects the organization of the NEXUS file format, as well as the implicit data models used in bioinformatics. For this reason, some information is stored literally, such as the order of blocks in a file or the taxa listed in a TaxaBlock, while some information is interpreted, such as the New Hampshire (a.k.a., Newick) tree string in a TreesBlock. The handling and placement of comments in NEXUS files is defined ambiguously by the standard. Bio::NEXUS keeps track of comment order and position relative to blocks, rather than relative to commands or the data they may contain. The exception is the case of tree strings, where square-bracketed strings are used to store node-associated data (by convention, branch-support or bootstrap values are stored as square-bracketed strings after nodes in a tree string; the NHX standard [13] described in the Discussion takes this convention further, allowing various types of data to be stored within square-bracketed strings within the tree string).

At the topmost level, a Bio::NEXUS object comprises an ordered list of block objects and block-level comments (comments found outside of blocks). Block objects may contain comments, simple attributes (e.g. alignment length and gap character in a CharacterBlock object), and objects (e.g. a Tree object within a TreesBlock object). Objects are used for data with complex structure or when data may require complex manipulations. Both are true of trees.

The organization of the Bio::NEXUS object is based on specific data models. A set of data is defined by a list of OTUs ("taxa" in the NEXUS language), or unique identifiers, provided in a TaxaBlock object. All other data provide either relationships between OTUs (e.g. trees, taxsets specifying sets of OTUs), or attributes of specific OTUs (e.g. protein sequences, intron positions). Some components of Bio::NEXUS objects are also based on specific data models. For example, Tree objects implicitly represent directed acyclic graphs, and therefore are represented by a hierarchy of Node objects with parent-child relationships. CharactersBlock objects are based on the character-state data model already described: each taxon ("entity") is stored as a TaxUnit object that has an associated sequence, or one state ("value") for each character ("attribute"). Characters that are polymorphic (denoted in a NEXUS file using parentheses, e.g. "TCA(AG)C") or unresolved (denoted using curly braces) may have multiple states for each character. If a frequency distribution is known for characters with multiple states, that is also stored.

## Results

### Examples of utility code: re-naming, re-rooting, and format conversion

Bio::NEXUS facilitates rapid development of utility scripts and "glue code" for typical bioinformatics tasks such as creating formatted files, manipulating data in files, converting formats, and developing wrappers. Below we provide several examples based on the file "example.nex" (see Additional file 2), which consists of the following text:

#NEXUS

BEGIN TAXA;

      DIMENSIONS ntax=4;

      TAXLABELS A B C D;

END;

BEGIN CHARACTERS;

      DIMENSIONS nchar=25;

      FORMAT DATATYPE=protein;

MATRIX

   A IKKGANLFKTRCAQCHTVEKDGGNI

   B LKKGEKLFTTRCAQCHTLKEGEGNL

   C STKGAKLFETRCKQCHTVENGGGHV

   D LTKGAKLFTTRCAQCHTLEGDGGNI

;

END;

BEGIN TREES;

      TREE my_tree = (((A:2,B:3):1,C:1):1,D:1)root;

END;

In the course of carrying out a scientific analysis, researchers will often wish to carry out complex operations that edit, transform or otherwise manipulate data stored in a file. While such steps often can be done "by hand" using a text editor, software tools reduce errors and allow automation. Bio::NEXUS facilitates developing glue code for these operations because, in addition to providing mutators and accessors for the elements of NEXUS objects, it also provides higher-level methods that perform a composite of operations or that carry out a non-trivial manipulation of data.

Re-rooting a tree is a common manipulation that typically is far too complex to carry out by editing a tree string directly with a text editor. In the example of re-rooting shown below, a Bio::NEXUS object is constructed, the manipulation is performed, and the altered object is written out to a new file.

use Bio::NEXUS;

my $nexus_obj = new Bio::NEXUS('example.nex');

$nexus_obj = $nexus_obj->reroot('A');

$nexus_obj->write('rerooted.nex');

Datasets can contain multiple trees, in which case this procedure would affect the default tree, unless a different tree is specified by name (in the NEXUS standard, each tree can have a name).

In the example of naming below, a Bio::NEXUS object is constructed, and a translation hash is defined. Single letter names are replaced with more meaningful names by calling the rename_otus method. Finally, the object is written out to a new file.

use Bio::NEXUS;

my $nexus_obj = new Bio::NEXUS('example.nex');

my %translate = ('A' => 'Xenopus laevis', 'B' => 'Mus musculus', 'C' => 'Pan paniscus', 'D' => 'Homo sapiens');

$nexus_obj->rename_otus(%translate);

$nexus_obj->write('renamed.nex');

Format conversion is a common problem in bioinformatics. Formats commonly encountered in evolutionary bioinformatics include PHYLIP, FASTA, MEGA, GCG (PileUp), and ClustalW. Although Bio::NEXUS does not does not include high-level methods that directly parse or output formats other than NEXUS, it simplifies format conversion considerably given that NEXUS is the most complex format commonly encountered. For instance, in the example below, the protein sequence alignment from example.nex is extracted and written out in FASTA format:

use Bio::NEXUS;

$nexus_obj = new Bio::NEXUS('example.nex');

$char_block = $nexus_obj->get_block('characters');

open(FASTA, '> example.fasta');

foreach $otu (@{ $char_block->get_otus()}){

      print FASTA ">", $otu->get_name(), "\n";

      print FASTA @{$otu->get_seq()},"\n";

}

In the example below of converting to the format used by the PHYLIP [14] package, an additional line of code is required to get and print the number of taxa (OTUs) along with the length of the alignment (using get_ntax and get_nchar). Also, inside the foreach loop that prints the alignment itself, a more complex print command is used to ensure that OTU names occupy no more than ten characters, as per the PHYLIP [14] standard (the new names become Pan_panisc, Xenopus_la, Homo_sapie and Mus_muscul).

use Bio::NEXUS;

$nexus_obj = new Bio::NEXUS('example.nex');

$char_block = $nexus_obj->get_block('characters');

open(PHYLIP, '> infile'); # "infile" is the filename PHYLIP requires

print PHYLIP $char_block->get_ntax(). ' ' . $char_block->get_nchar() . "\n";

foreach my $otu (@{ $char_block->get_otus() }) {

      printf PHYLIP "%-10.10s ", $otu->get_name();

      print PHYLIP @{$otu->get_seq,()}, "\n";

}

By calling additional methods, more complex scripts can be developed. For instance, the restriction on name lengths in PHYLIP files may create a problem if the truncated names are non-unique (e.g., the first 10 characters of Anolis_tubA and Anolis_tubB are the same). The problem of preserving long names while using PHYLIP programs can be solved by writing a Bio::NEXUS wrapper that uses NEXUS as its input and output format: internally the wrapper would use rename_otus to convert OTU names to a temporary set of short identifiers (e.g., otu_1, otu_2, . . .), interface with the PHYLIP package, add the PHYLIP-generated results to the NEXUS object, then use rename_otus again to convert back to the original names before writing out the modified object to a new NEXUS file.

### Example of a stand-alone tool: nextool.pl

From the lack of available software tools and the high frequency of non-compliant files in the user community, it is apparent that NEXUS users typically manage and edit their data files manually using a text editor. However, this is not practical for larger data sets and for large numbers of files. For instance, to re-root a tree by manipulating the tree string directly with a text editor is a daunting task that invites human errors. Safe removal of an OTU is equally daunting as it is not merely a matter of searching for the OTU label and deleting it along with any associated data. As the NEXUS standard allows taxa to be referenced by a number representing the order in which they were declared, the data for an OTU are not always associated syntactically with the OTU label; for the same reason, removing an OTU changes the system of referencing for all subsequent OTUs. Furthermore, if the OTU is the first shown in a character matrix, then--following a convention widely used with sequence alignments – its character states may be used as a reference states such that, for other OTUs listed below it, only differences are shown (with similarities indicated typically by the "matchchar" symbol, typically a "."). In such cases, deleting the OTU is not possible without semantic processing and rewriting of the character matrix.

Indeed, the burden imposed by the complexity of the NEXUS format coupled with the lack of suitable editing tools may be one reason for the under-utilization of NEXUS files.

To address this problem, we have developed nextool.pl, a script that automates various tasks typically involved in managing, curating and editing data sets in NEXUS format. The nextool.pl script is found in the exec/directory of the Bio::NEXUS package, and is typically installed in the user's executable path during an automated installation. It operates as a command editor with the syntax:

nextool.pl <input_file> [<output_file>] [<command> [<arguments>]]

That is, an output file is generated by carrying out some command on the contents of the input file. Useful commands include **rewrite **(to rewrite a file in standard format), **rename_otus **(using, as a command argument, a string that maps old names to new ones, or the name of a file with such a mapping), **reroot **(to reroot a tree on a named node or OTU), **select**, **exclude **and **makesets**. The **select **and **exclude **commands may operate on blocks, columns (characters), trees, subtrees or OTUs. For instance, the command

nextool.pl. infile outfile select columns "1–3, 8, 10–45"

creates an output file "outfile" with only columns (characters) 1–3, 8, and 10–45 selected from the input file "infile". The command

nextool.pl infile outfile makesets bycharstate "isotype A"="124 H"

would add an OTU set named "isotype A" including only those OTUs that have the character state "H" for character number 124. This named set then could be referenced in subsequent operations such as **select **or **exclude**. Sets can also be referenced in set operations, so that (for instance), we could define a set consisting of all OTUs that have "H" for character 124 but do not have "E" for character 271.

### Example of a web-based application: Nexplorer

Bio::NEXUS plays a key role in Nexplorer, a web-based program for interactive browsing and manipulation of character state data and trees [15]. Nexplorer has a 3-tiered design: the front end consists of JavaScript, HTML and images, the back end consists of NEXUS files along with a database of taxonomic information, and the middle layer consists of CGI and Bio::NEXUS, which is used to access and manipulate the data in NEXUS files. To generate custom views of data or to create subsets of data for further analysis, users need only to upload a properly formatted NEXUS file to Nexplorer. The server responds by generating an image of the data and by mapping such things as JavaScript pop-up menus to the nodes of tree that allow the user to re-root a tree, exclude or select a subtree, and so on.

## Discussion and conclusion

### Comparison with other implementations

As Lewis [12] suggests, developing a library to support NEXUS fully is difficult due to the flexibility built into the standard. According to Maddison et al. [4] writing in 1997, "no program can understand more than about 60% of the elements described" by the standard.

To measure the degree of implementation precisely, we have used the Backus-Naur formalization of NEXUS provided by Iglesias, et al. [16] to generate a list of keywords. The keywords are reserved tokens representing commands and subcommands (or command modifiers), some of them (e.g., "interleave") used in several different blocks. The total number of keyword-block combinations is 119. Currently Bio::NEXUS provides some support for 80% (8 of 10) of the block types (all but NOTES and CODONS) and 68% (81/119) of the keyword-block combinations. The latter number is reflected in the current version number (0.68) of the Bio::NEXUS release.

Other currently available software libraries to support NEXUS include NCL [12] and the code available in various Bio* toolkits [17]. According to the documentation, NCL implements TAXA, TREES and DISTANCES completely, and implements CHARACTERS and ASSUMPTIONS partially. Other implementations typically focus on TAXA and TREES block, and support common uses of CHARACTERS blocks without implementing various format options. Bio::Phylo by Rutger Vos [18] focuses on TAXA, TREES and CHARACTERS. BioPerl supports TAXA, TREES, and CHARACTERS blocks, but writes data only in the deprecated DATA block format. The BioJava NEXUS parser handles DATA, CHARACTERS, DISTANCES, TREES, and TAXA blocks. The Mesquite project [5], also in Java, provides a documented NEXUS API with support for all standard blocks except UNALIGNED.

### Extensions to the NEXUS standard

Bio::NEXUS implements a few extensions to the NEXUS standard. One of these is the convention used in Mesquite project files of allowing multiple blocks of the same type, with linking-by-name between blocks. The **title **command is used to assign a name to a block, and the **link **command is used to refer to another block by its title, e.g., a TREES block with trees inferred from the data in a CHARACTERS block entitled "gene data" could have the command

   link characters = "gene data";

Bio::NEXUS also implements the NHX (New Hampshire Extended) standard of Zmasek and Eddy [13]. NHX expands the original "Newick" or "New Hampshire" standard, wherein the clades of a phylogeny are represented by pairs of nested parentheses (see the examples in Results above). NHX tree strings incorporate special NEXUS comments that contain tags specifying additional information about nodes of a tree. The syntax of an NHX comment is [&&NHX:<tag>=<value>], the comment follows the node, and each tag is responsible for carrying specific information about the given node. For example, the "B" tag refers to the branch support (e.g., bootstrap) value at the given node, and "T" tag refers to the NCBI taxonomy ID. The Bio::NEXUS::NHXCmd module provides the support for reading, manipulation, and writing of the NHX comments. Methods in the Bio::NEXUS::Node class provide the capacity to add, remove, and edit specific NHX tags and the corresponding values.

### Future challenges for NEXUS, Bio::NEXUS and evolutionary informatics

NEXUS files have been in use for many years. Users and developers presumably choose NEXUS because it is the preferred or required input file for valuable programs (e.g., PAUP*), because of the capacity to store trees with data, and because of the expressivity that allows diverse kinds of data to be represented along with constraints or assumptions. As integrative and evolutionary approaches to biological analysis become more common, there will be a growing need for formats that provide the functionality of NEXUS.

Yet serious problems have emerged from the complexity of the standard and the historical lack of software support. First, the standard is so complex that users and developers commonly misunderstand it (a substantial fraction of NEXUS files in current use violate the standard; several software applications generate or expect poorly formed or deprecated file formats). Because there is no generalized NEXUS editor, users apparently hand-edit their files using text editors or word processors, leading to typographic errors and to poorly formed files. Second, the standard is so extensive, with so many options and so many variations in syntax, that no developer has attempted a complete implementation, except for the developers of a Prolog-based parser [16]. Indeed, some applications support only the deprecated DATA block format. Finally, the NEXUS grammar is more like that of a natural language and cannot be parsed using conventional scanning-parsing routines, due to the inability to resolve ambiguous tokens prior to extensive semantic processing [16]. The next-generation standard to replace NEXUS should be defined in a formal language, and it should i) be more easily parsed; ii) make use of available technology that facilitates crucial tasks such as validation and editing (e.g., as for an XML-based standard such as PhyloXML [19]); iii) import ontologies for character data (e.g., nucleotide or amino acid states); and perhaps iv) utilize a process-specification language to describe steps in an analysis (both previous steps and subsequent ones to be carried out).

Nevertheless, as there is no replacement currently on the horizon, it seems clear that NEXUS files will continue to be used for years to come. A robust and easy-to-use API such as Bio::NEXUS will make it easier to manage NEXUS files, to maintain legacy data and convert it to other forms, and– when combined with greater community attention to standards for integrative analyses – will facilitate the transition to future technologies to achieve integration. Future plans for Bio::NEXUS, intended to facilitate the ongoing use of NEXUS and to protect legacy data, include offering 100 % support for the standard and common extensions, validation capacity, and object integration with BioPerl [11].

## Availability and requirements

Bio::NEXUS requires Perl 5.x, available for free download from CPAN [20]. The current release of Bio::NEXUS is available as an installable package from CPAN [20]; the current development version of the source code is available from SourceForge [21]. The CPAN package includes Perl code, test files, documentation, some useful scripts (e.g., nextool.pl), and examples. On UNIX-like systems (Mac OS X, Linux, UNIX, or Cygwin running within Windows) Bio::NEXUS can be installed using "perl -MCPAN -e 'install Bio::NEXUS"'; or on Windows systems using ppm (Perl Package Manager). The test suite (implemented with Perl's "Test" module) comprises over 30 test scripts implementing over 460 subtests. The documentation, written in POD (Plain Old Documentation) format, includes a user manual and a tutorial (see below for examples). The code files also are annotated with POD, hence references for Bio::NEXUS modules and their methods can be generated on the fly using the standard perldoc command.

## Authors' contributions

TJH helped to design and implement the code, to write the documentation, and to draft the manuscript; CL and WQ helped to design and implement early versions of the library and tools; PY and VG carried out testing and aided in implementation; AS participated in design, implementation and testing, helped to draft the manuscript, and oversaw all aspects of the project; all authors read and approved the manuscript.

## Supplementary Material

Additional file 1 figure1.nex. The NEXUS file corresponding to Figure 1. Click here for file

Additional file 2 example.nex. A simple NEXUS file used in the tutorial examples. Click here for file
